# Identification of Pro-Fibrotic Macrophage Populations by Single-Cell Transcriptomic Analysis in West Highland White Terriers Affected With Canine Idiopathic Pulmonary Fibrosis

**DOI:** 10.3389/fimmu.2020.611749

**Published:** 2020-12-15

**Authors:** Aline Fastrès, Dimitri Pirottin, Laurence Fievez, Alexandru-Cosmin Tutunaru, Géraldine Bolen, Anne-Christine Merveille, Thomas Marichal, Christophe J. Desmet, Fabrice Bureau, Cécile Clercx

**Affiliations:** ^1^ Department of Clinical Sciences, Faculty of Veterinary Medicine, Fundamental and Applied Research for Animals & Health (FARAH), University of Liège, Liège, Belgium; ^2^ Laboratory of Cellular and Molecular Immunology, Department Functional Sciences and GIGA-Inflammation, Infection & Immunity, University of Liège, Liège, Belgium

**Keywords:** macrophages, canine idiopathic pulmonary fibrosis, dog, single-cell RNA-sequencing methods, bronchoalveolar lavage fluid, lung

## Abstract

Canine idiopathic pulmonary fibrosis (CIPF) affects old dogs from the West Highland white terrier (WHWT) breed and mimics idiopathic pulmonary fibrosis (IPF) in human. The disease results from deposition of fibrotic tissue in the lung parenchyma causing respiratory failure. Recent studies in IPF using single-cell RNA sequencing (scRNA-seq) revealed the presence of profibrotic macrophage populations in the lung, which could be targeted for therapeutic purpose. In dogs, scRNA-seq was recently validated for the detection of cell populations in bronchoalveolar lavage fluid (BALF) from healthy dogs. Here we used the scRNA-seq to characterize disease-related heterogeneity within cell populations of macrophages/monocytes (Ma/Mo) in the BALF from five WHWTs affected with CIPF in comparison with three healthy WHWTs. Gene set enrichment analysis was also used to assess pro-fibrotic capacities of Ma/Mo populations. Five clusters of Ma/Mo were identified. Gene set enrichment analyses revealed the presence of pro-fibrotic monocytes in higher proportion in CIPF WHWTs than in healthy WHWTs. In addition, monocyte-derived macrophages enriched in pro-fibrotic genes in CIPF compared with healthy WHWTs were also identified. These results suggest the implication of Ma/Mo clusters in CIPF processes, although, further research is needed to understand their role in disease pathogenesis. Overexpressed molecules associated with pulmonary fibrosis processes were also identified that could be used as biomarkers and/or therapeutic targets in the future.

## Introduction

Canine idiopathic pulmonary fibrosis (CIPF) is defined as a progressive and abnormal accumulation of collagen in the lung parenchyma that threatens alveolar gas exchange and reduces lung compliance causing cough, exercise intolerance, and, finally, respiratory failure and death (1, 2). The disease affects predominantly middle-aged to old dogs from the West Highland white terrier (WHWT) breed (1, 2). Although the cause of CIPF is not identified, a genetic etiology is suspected as it affects mainly one breed. Confirmation of the diagnostic remains challenging due to absence of available diagnostic biomarkers and necessity to exclude other diseases and comorbidities. It currently relies on either thoracic high-resolution computed tomography (HRCT) or histopathology of the lung tissue or both. Despite a lot of researches on CIPF, the pathophysiology remains unclear and no curative treatment are available (1, 2).

CIPF shares several clinical findings with human idiopathic pulmonary fibrosis (IPF). However, thoracic HRCT and histopathology show features associated with both human IPF and non-specific interstitial pneumonia demonstrating that CIPF and IPF are not strictly identical (1, 2). In spite of those differences, studying CIPF in WHWTs is worth to better understand IPF. Indeed, dogs, like human, are subjected to various environmental stresses which can have an impact on lung cells especially alveolar macrophages (AMs) (3). Moreover, CIPF is a disease that develops spontaneously in WHWTs (1, 2). Those characteristics make the dog a much more interesting model compared to the mouse experimental models. In human IPF and IPF mouse models, recent studies used single-cell mRNA sequencing (scRNA-seq) to detect altered cell populations compared with healthy conditions through an unbiased approach (4–13). Indeed, the technique allows high-throughput and high-resolution analysis of thousands of cells at the same time without requiring prior knowledge of cell markers to determine cell heterogeneity (14–16). With this method, a profibrotic role of specific macrophage and monocyte populations has been described in IPF patients and IPF mouse models (4, 6, 7, 12, 17). An increased number of macrophages and proliferating myeloid cells was found in bleomycin-induced lung fibrosis mouse models, in the beginning of lung fibrosis development, before fibroblastic infiltration (6). Specific monocyte and macrophage clusters were identified in fibrosis conditions (4, 5, 7, 12). AMs from IPF patients were enriched in functions involved in fibrotic processes including “extra-cellular matrix organization” and “regulation of cell migration” for example (5). Pro-fibrotic macrophage but also monocyte clusters that expressed genes able to drive fibroblasts’ proliferation were localized in areas of fibrosis (4, 12). All these findings indicate that targeting specific macrophage and monocyte clusters could be potentially useful for the prevention and the therapy of lung fibrosis (17).

Recently, cells of the bronchoalveolar lavage fluid (BALF) of healthy dogs have been characterized by scRNA-seq, providing a comprehensive single-cell expression profiling of the canine BALF cells in healthy conditions (18). Fourteen distinct cell populations were identified including AMs (three clusters), macrophages/monocytes (Ma/Mo) (one cluster), CD8^+^ T cells, CD8^−^CD4^−^ T cells, B cells, neutrophils, mature and immature dendritic cells (DCs), ciliated and non-ciliated epithelial cells, mast cells, and cells in division (18).

The objective of this study was to characterize, using scRNA-seq, disease-related heterogeneity within Ma/Mo populations in the BALF from WHWTs affected with CIPF compared with healthy WHWTs.

## Materials and Methods

### Dog Population

The scRNA-seq analysis was performed on BALF obtained from WHWTs affected with CIPF and healthy WHWTs. Dogs were prospectively recruited between March and October 2018 at the veterinary clinic of the University of Liège (Liège, Belgium) according to a protocol approved by the ethical committee of the University of Liège (approval no. 1435). All dogs were privately owned, and samples were obtained with owners’ written consent.

The healthy or CIPF status of the dogs was confirmed according to a previously described approach (19) based on history, physical examination, complete blood work, 6-min walked distance (6MWD), thoracic HRCT, bronchoscopy, and analysis of the BALF (including macroscopic evaluation and total (TCC) and differential (DCC) cell count). WHWTs under treatment including antimicrobials drugs and corticoids were excluded from the study.

### BALF Collection

BALF was obtained using the same protocol as previously described (18). Briefly, under general anesthesia, a bronchoscope (FUJINON© Paediatric Video-Bronchoscope EB-530S) was inserted into the lower airways of the dogs. Three to 4 ml/kg of sterile saline solution was instilled in the airways through the bronchoscope channel and directly reaspirated. A part of the crude BALF was used for TCC and DCC obtained using respectively a hemocytometer and a cytospin preparation. The rest of the collected fluid was then directly transferred on ice to the GIGA laboratory of cellular and molecular immunology (Liège, Belgium).

### Single-Cell RNA Sequencing

ScRNA-seq was performed as already described (18, 20). Briefly, BALFs were processed to obtain a final cell suspension containing between 500 and 1,000 cells/µl suspended in phosphate-buffered saline solution (Gibco^TM^ 1x DPBS, Cat.14190-169) containing 0.04% (w/v) bovine serum albumin. Cell viability assessed by Trypan blue staining was considered as acceptable above 80%. Details about BALF volume, final cell concentration and cell viability for each sample can be found in **Supplementary Table 1**.

For each sample, approximatively 3,500 cells (**Supplementary Table 1**) were loaded into the Chromium^TM^ Controller (10x Genomics, Pleasanton, CA, USA) and were then partitioned into nanoliter scale vesicles containing 10x barcoded beads from Chromium^TM^ Single Cell 3’ Gel Bead kit v2 (10x Genomics, Pleasanton, CA, USA) according to manufacturer’s instructions. Reverse transcription of mRNAs took place into vesicles on a Veriti© 96-Well Thermal Cycler (ThermoFisher Scientific, Merelbeke, Belgium) after cell lysis and capture of polyadenylated mRNAs.

Emulsion breakage, cDNA amplification, and libraries construction were performed using Chromium^TM^ Single Cell 3′ Reagent kit v2 (10x Genomics, Pleasanton, CA, USA) according to manufacturer’s instructions as already described (18, 20). Libraries were assessed for quality (2100 Bioanalyser Instrument; Agilent, Santa Clara, CA, USA) and then sequenced on a NextSeq500 instrument (Illumina, San Diego, CA, USA).

Initial data pre-processing was performed using the Cell Ranger software (v1.2.0) (10x Genomics, Pleasanton, CA, USA). Reads were mapped to dog genome (CanFam3.1, GenBank assembly accession: GCA_000002285.2). The genes not well annotated were further blasted on the Ensembl genome browser (v99.31) (21) for dog species.

Further data analyses were performed using R package Seurat (version 3.1.2) (22) after the selection of the cells with a minimum of 100 and a maximum of 2,500 unique genes mapped, the selection of the genes found in at least three different cells and the normalization of the expression values to 10,000 transcripts per cell. ScRNA-seq data coming from each dog were then merged for the next analyses which were done by following Stuart et al. (2019) instructions (22). Pre-ranked gene set enrichment analyses (GSEAs) were performed using GSEA-P software (v4.0.3) (23). The enrichment score was determined using weighted Kolmogorov–Smirnov-like statistic with false discovery rate (FDR) correction for multiple testing (23). A FDR cutoff of 25%> was considered as appropriate (23). GSEAs were computed between either the Gene Ontology (GO) Biological Process gene sets (v7.1) (23), or the Hallmark gene sets (v7.1) (23) or the Comparative Toxicogenomics Database Pulmonary fibrosis gene set (24). Differentially expressed genes (DEGs) in different conditions were obtained using the “FindMarkers” command in Seurat (22). Differential gene expressions were measured using non-parametric Wilcoxon rank sum tests adjusted for multiple testing with Bonferroni correction. Only DEGs with an average log2 fold change (avg_logFC) >0.25 and an adjusted *P-*value <0.05 were retained.

### Statistical Analyses

A P-value lower than 0.05 was considered as significant. Details about statistical analyses for scRNA-seq data and GSEAs can be found in the section above. Statistics used for the comparison of the WHWTs groups are reported in **Tables 1**, **3**, and **4**.

**Table 1:** Characteristics of the West Highland white terriers either healthy or affected with canine idiopathic pulmonary fibrosis included in the study.

		Healthy WHWTs (n = 3)	WHWTs affected with CIPF (n = 5)	*P*-value
Age, *y*		8.2 (5.4–8.7)	10.8 (10.2–12.7)	0.14^a^
Gender, *M/F*		2/1	1/4	0.46^b^
Weight, *kg*		8.4 (8.4–8.9)	9.5 (9.1–9.9)	0.14^a^
6MWD, *m*		506.1 (478.8–513.0)	356.4 (356.1–366.3)	0.04^a^
BALF analysis	TCC, *cells/µl*	760 (665–770)	2,620 (2,500–3,285)	0.04^a^
	Macrophages, *%*	78 (76.5–84.5)	71 (64–82)	0.39^a^
	Neutrophils, *%*	3 (2.5–3.5)	10 (9–21)	0.04^a^
	Lymphocytes, *%*	11 (7–16)	7 (7–16)	0.93^a^
	Eosinophils, *%*	1 (1–4)	2 (1–2)	0.93^a^
	Mast cells, *%*	0	0	/
	Epithelial cells, *%*	1 (0.5–1.5)	1 (0–1)	0.46^a^

Continuous data are not normally distributed according to the Shapiro-Wilk test and are then expressed in median and interquartile range. Groups were compared using either Mann-Whitney tests (^a^) or Chi-squared tests (^b^). WHWTs, West Highland white terriers; CIPF, canine idiopathic pulmonary fibrosis; M, male; F, female; 6MWD, 6-min walked distance; BALF, bronchoalveolar lavage fluid; TCC, total cell count.

## Results

### Study Population

BALF samples were obtained from three healthy WHWTs and five WHWTs affected with CIPF. Characteristics of the dogs included in the study are reported in **Table 1**. No significant differences in age, gender, and weight were reported between the groups (**Table 1**).

CIPF diagnosis was confirmed in all CIPF WHWTs by thoracic HRCT which revealed extensive ground-glass opacity in all dogs. Other HRCT findings included a combination of mosaic pattern, bronchial wall thickening, parenchymal and subpleural bands, bronchomalacia and bronchiectasis. Among WHWTs affected with CIPF, 3/5 (60%) had an history of both exercise intolerance and cough and 2/5 (40%) only exhibited exercise intolerance. Crackles were heard on lung auscultation in all dogs. Three dogs (60%) had a restrictive dyspnea. Among them, two also exhibited cyanosis. The 6MWD covered by each dog was in favor of exercise intolerance in all CIPF dogs. Moreover, the distance was significantly reduced in CIPF compared with healthy WHWTs (**Table 1**). At echocardiography, signs of secondary pulmonary arterial hypertension were present in all CIPF dogs. Changes in BALF cells analysis were consistent with non-specific chronic lung inflammation (**Table 1**).

Among control WHWTs included in the study, all were clinically healthy and did not have any signs or findings indicating pulmonary disease. Echocardiography excluded the presence of cardiac disease in all of them. Thoracic HRCT did not reveal significant abnormalities. BALF cells analysis was unremarkable (**Table 1**).

### ScRNA-Seq Identifies Multiple Cell Populations in the Dog BALF

Droplet-based scRNA-seq analysis of BALF cells was performed with a median read depth of ∼43,000 reads per cell. In total, 19,255 cells (6,703 from healthy and 12,552 from diseased dogs) coding for 11,722 unique genes were included in the final analysis. The median detected genes per cell was 788 (interquartile range 399–1,191 genes/cells, **Table 2**). Individual metrics about mapping and cells are displayed in **Table 2**, the individual distribution for transcripts and genes counts is illustrated in **Supplementary Figures 1A and B**, respectively.

**Table 2:** Metrics about mapping and characteristics of the detected cells in each bronchoalveolar lavage fluid specimen.

Sample ID	Diagnosis	Number of cells passing quality control	Reads mapped confidently to genome, *%*	Reads mapped confidently to transcriptome, *%*	Median genes/cell (range)	Median UMIs/cell (range)	Total genes detected
WHWT 1	Healthy	3,060	67	24.4	741 (445–1,184)	1,711 (899–3,351)	12,354
WHWT 2	Healthy	2,345	67.2	24.7	1,091 (585–1,446)	2,809 (1,173–4,398)	12,988
WHWT 3	Healthy	1,298	59.8	23.9	834 (376–1,046)	1,889 (678–2,671)	10,839
CIPF 1	CIPF	2,551	69.2	24.6	1,147 (827–1,346)	2,934 (1,857–3,740)	12,988
CIPF 2	CIPF	2,686	67.3	23.4	618 (219–1,226)	1,362 (354–3,390)	12,478
CIPF 3	CIPF	2,564	74.8	30.4	503 (355–969)	960 (601–2,247)	11,819
CIPF 4	CIPF	2,556	71.5	28.1	867 (411–1,090)	1,939 (708–2,754)	11,722
CIPF 5	CIPF	2,195	73.1	27.5	453 (383–722)	833 (656–1,622)	11,921

Data were generated after passing quality control including the exclusion of cells with <100 and >2,500 genes. Only genes present in more than three cells were kept. Reads mapped confidently to genome are the number of reads that mapped only to the genome. Reads mapped confidently to transcriptome are the fraction of the reads mapped to a unique gene in the transcriptome and are considered for UMI counting. Median genes per cell correspond to the median number of genes with at least one UMI count. Total genes detected is the detected number of genes with at least one UMI count in any cell. ID, identity; UMI, unique molecular identifier; WHWT, West Highland white terrier; CIPF, canine idiopathic pulmonary fibrosis.

**Figure 1:**
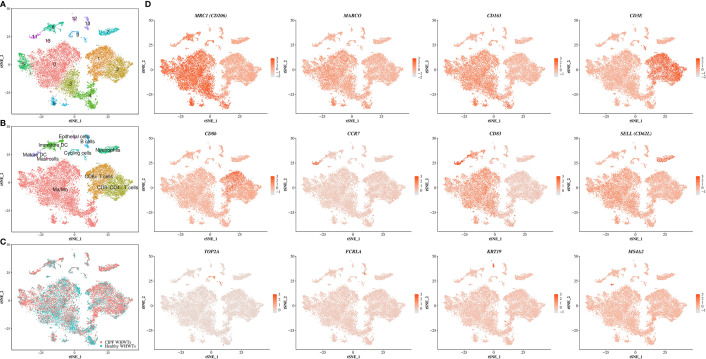
Single-cell RNA sequencing analysis identifies multiple cell populations in the canine bronchoalveolar lavage fluids (BALFs). The scRNA-seq analysis was performed on a single-cell suspension generated from eight BALFs obtained from three healthy West Highland white terriers (WHWTs) and five WHWTs affected with canine idiopathic pulmonary fibrosis (CIPF). Cells were visualized using t-distributed stochastic neighbor embedding (t-SNE) plots. **(A)** Cell clusters identified. **(B)** Cell populations identified. **(C)** Cells are colored according to the status of dogs either healthy or affected with CIPF. **(D)** Expression of differentially expressed genes representative of each cell population. Ma/Mo, macrophages/monocytes; DC, dendritic cell; *MRC1*, macrophage mannose receptor; *MARCO*, macrophage receptor with collagenous structure; *CD163*, scavenger receptor cysteine-rich type 1 protein M130; *CD3E*, T-cell surface glycoprotein CD3 epsilon chain; *CD8b*, T-cell surface glycoprotein CD8 beta chain; *CCR7*, C-C chemokine receptor type 7; *CD83*, CD83 molecule; *SELL*, selectin; *TOP2A*, DNA topoisomerase II alpha; *FCRLA*, Fc receptor like A; *KRT19*, cytokeratin 19; *MS4A2*, membrane spanning 4-domains A2.

Cells from all samples were combined and aligned to account for sample variations among dogs using Seurat package in R (version 3.1.2) (22). They were then clustered and visualized using t-distributed stochastic neighbor embedding (t-SNE) plot with a resolution set at 0.3 and a number of dimensions to use set to 30 which resulted in the identification of 14 clusters (**Figure 1A**). After clustering, DEGs between each identified cluster were used to assign cell types to each cluster using previously established markers (18). Cells populations found accordingly included Ma/Mo (5 clusters), CD8^+^ and CD8^−^CD4^−^ T cells, mature and immature DCs, neutrophils, B cells, epithelial cells, mast cells and cycling cells (**Figure 1B**). DEGs detected in each cluster are provided in **Supplementary Table 2**. Principal markers used to identify cell populations can be found in **Figure 1D**. Each cell population included cells coming from healthy and diseased dogs (**Figure 1C** and **Table 3**). No significant differences were reported in relative proportions of the different cell types between healthy WHWTs and WHWTs affected with CIPF, except for mature DCs (**Table 3**).

**Table 3:** Relative cells repartition between healthy and CIPF WHWTs in each cell population.

	Healthy WHWTs	CIPF WHWTs	*P*-value
Ma/Mo	69.5 ± 4.7	52.7 ± 26.3	0.332
CD8^+^ T cells	10.9 ± 10.0	17.6 ± 15.4	0.533
CD8^-^CD4^-^ T cells	7.1 ± 1.1	14.7 ± 9.0	0.210
Immature DC	4.6 ± 3.1	3.7 ± 1.6	0.586
Neutrophils	1.6 ± 2.1	4.5 ± 6.7	0.498
Cycling cells	2.2 ± 0.5	1.8 ± 0.7	0.456
B cells	1.5 ± 0.6	2.1 ± 1.4	0.557
Mature DC	0.4 ± 0.1	2.1 ± 1.0	0.041
Epithelial cells	1.0 ± 0.6	0.6 ± 0.2	0.160
Mast cells	1.2 ± 1.8	0.3 ± 0.3	0.270

Relative cell proportion were compared between healthy West Highland white terriers (WHWTs) and WHWTs affected with canine idiopathic pulmonary fibrosis (CIPF) using t-tests after verification of the distribution normality using Shapiro-Wilk tests. Data are expressed in mean percentage ± standard deviation.

### ScRNA-Seq Analysis Reveals Fibrosis-Associated Transcriptomic Changes in Ma/Mo Clusters

#### Comparison Between Ma/Mo Clusters

**Figure 2:**
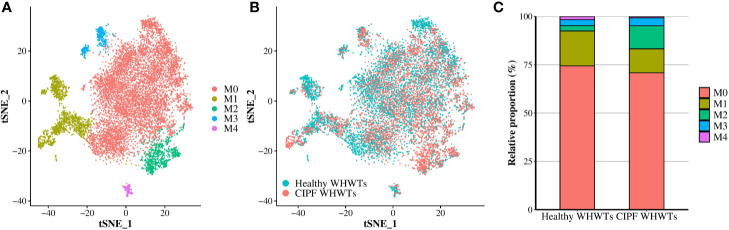
Macrophages/monocytes (Ma/Mo) clusters identified. Cells identified as Ma/Mo after the annotation of scRNA-Seq data obtained from three healthy West Highland white terriers (WHWTs) and five WHWTs affected with canine idiopathic pulmonary fibrosis (CIPF) were selected and then clustered allowing the identification of five distinct clusters. **(A)** Clusters identified. Cells were visualized using a t-distributed stochastic neighbor embedding (t-SNE) plot. **(B)** t-SNE plot of Ma/Mo colored according to the disease status of the WHWTs either healthy or affected with CIPF. **(C)** Bar plot of the relative proportion in each disease status of each Ma/Mo cluster.

After Ma/Mo isolation from other cell populations, we repeated clustering on those cells to better characterize changes associated with CIPF. It resulted in the identification of five transcriptionally distinct Ma/Mo clusters (M0, M1, M2, M3, and M4) (**Figure 2A**). Average expression of all the genes expressed by each Ma/Mo cluster can be found in **Supplementary Table 3**. Relative contributions of each Ma/Mo cluster into each group of dogs either healthy or diseased are displayed in **Figures 2B, C**. Cells repartition between healthy and diseased WHWTs was similar into each cluster except in the cluster M2 which contained more cells in WHWTs affected with CIPF (**Figures 2B, C** and **Table 4**). We then estimated differential genes expression between each cluster of Ma/Mo and performed GSEAs to better characterize Ma/Mo clusters independently of the disease status of the dogs. All DEGs identified in each cluster compared to others are displayed in **Supplementary Table 4**. Results of the enrichment analyses performed by mapping DEGs identified in each cluster compared to others, to Hallmark gene sets or GO Biological Process gene sets are provided in **Supplementary Table 5**.

**Table 4:** Relative cells repartition between healthy and CIPF WHWTs in each Ma/Mo cluster.

	Healthy WHWTs	CIPF WHWTs	*P*-value
M0	73.6 ± 4.1	67.2 ±10.0	0.342
M1	18.8 ± 4.1	14.2 ± 6.6	0.329
M2	2.9 ± 0.2	13.5 ± 4.7	0.009
M3	3.2 ±1.1	4.4 ± 1.8	0.356
M4	1.4 ± 1.0	0.7 ± 0.4	0.185

Relative cell proportion in each macrophages/monocytes (Ma/Mo) cluster were compared between healthy West Highland white terriers (WHWTs) and WHWTs affected with canine idiopathic pulmonary fibrosis (CIPF) using t-tests after verification of the distribution normality using Shapiro-Wilk tests. Data are expressed in mean percentage ± standard deviation.

**Figure 3:**
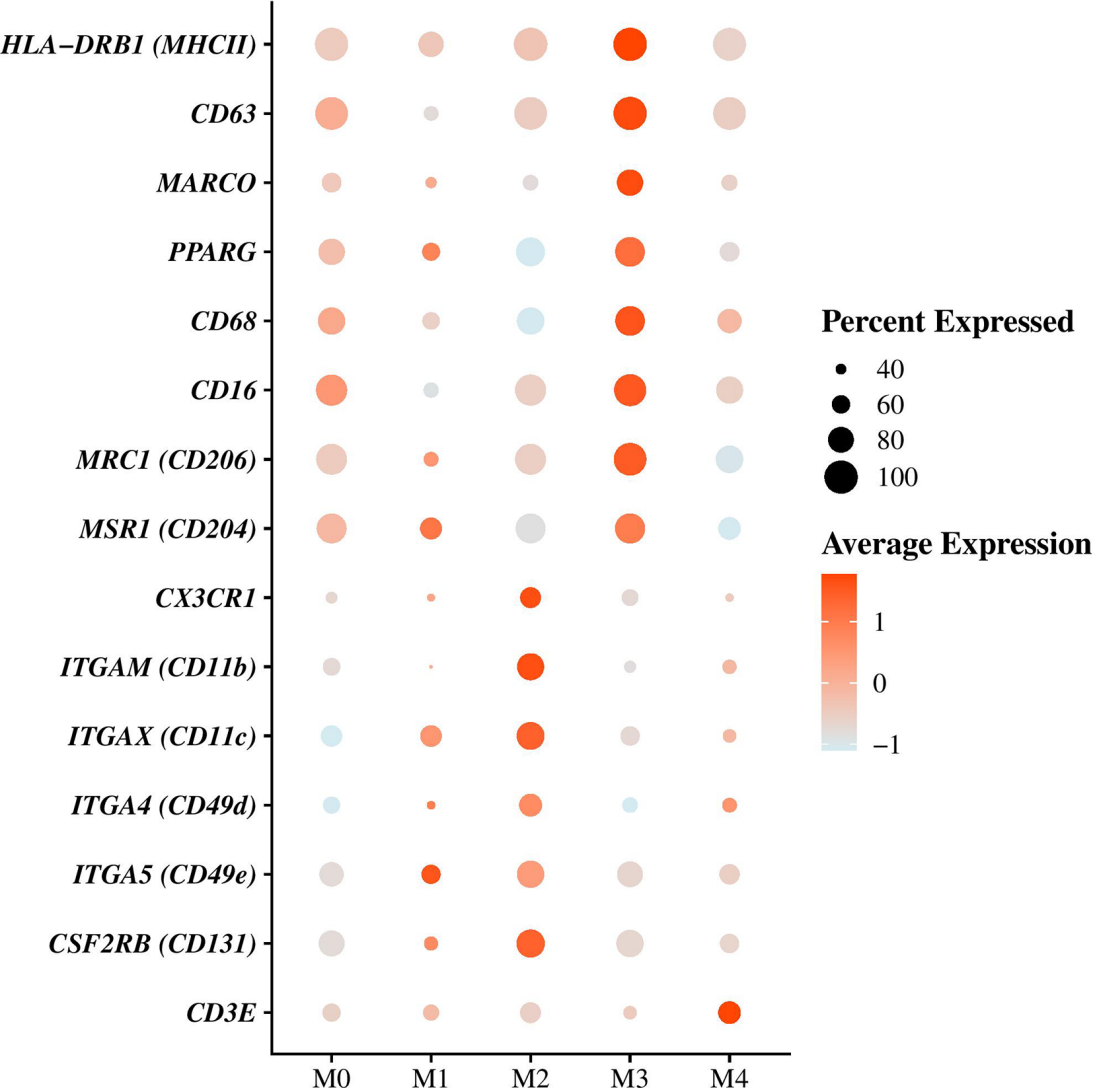
Differential genes expression analysis between macrophages/monocytes (Ma/Mo) clusters. Dot plot showing the expression of the principal gene markers used to characterize each Ma/Mo cluster. Dot size represents the percentage of cells expressing the genes, while the dot color represents the average expression of the indicated genes.

Resident AMs were identified based on *MARCO* expression (**Figure 3**), a class A scavenger receptor (5, 12, 18, 25, 26) and corresponded to cells in clusters M0 and M3 (**Figure 2A**, **Supplementary Tables 3** **and** **4**). They represented the majority of the cells composing Ma/Mo population (**Figures 2A, C** and **Table 4**). Cells in these clusters were enriched in biological processes relevant to AMs including “Hallmark reactive oxygen species pathways” for M0 cells and “GO adaptative immune response,” “GO antigen processing and presentation of peptide or polysaccharide antigen via MHC class II,” “GO activation and regulation of immune response,” and “GO pattern recognition receptor signaling pathway” for M3 cells (**Supplementary Table 5**). Cells in cluster M1 were considered as monocyte-derived macrophages as they expressed markers from both macrophages, including *MARCO*, *PPARG* (encoding peroxisome proliferator activated receptor gamma), *CD68*, *MRC1* (encoding macrophage mannose receptor, *CD206*), *MSR1* (encoding macrophage scavenger receptor 1, *CD204*) and *CD16* (5, 27–31), and monocytes, including *CD11c* (encoding integrin subunit alpha X, *ITGAX*), *CD16*, *CD49d* (encoding integrin subunit alpha 4, *ITGA4*), *CD49e* (encoding integrin subunit alpha 5, *ITGA5*) and *CX3CR1* (encoding fractalkine receptor) (**Figure 3**, **Supplementary Tables 3** and **4**) (29, 30, 32, 33). A cluster of monocytes which corresponded to cluster M2 was also identified. Indeed, M2 cells expressed only monocytes markers (**Figure 3**) including *CSF2RB* (encoding colony stimulating factor 2 receptor subunit beta, *CD131*)*, CD11c*, *CD11b* (encoding integrin subunit alpha M, *ITGAM*), *CD49d*, *CD49e*, and *CX3CR1* (**Figure 3**, **Supplementary Tables 3** and **4**) (29, 30, 32–34). Cells in cluster M1 were enriched in functions associated with the immune response activation including “Hallmark inflammatory response,” “GO interferon gamma production,” and “GO leukocyte cell-cell adhesion,” while cells in cluster M2 were more involved in “GO leukocyte migration” and “GO cell motility” (**Supplementary Table 5**), functions essential when monocytes are recruited from blood into tissues. M4 cells also expressed macrophages and monocytes markers including notably *MHC-II* markers, *CD63, CD68, CD16*, and *CD49d* (12, 30, 32, 33, 35), but they also overexpressed *CD3* genes compared with other clusters (**Figure 3** and **Supplementary Tables 3** and **4**) which are known to be T-lymphocyte markers (36). Enriched processes associated with M4 cluster were mainly focused on inflammatory response (**Supplementary Table 5**).

**Figure 4:**
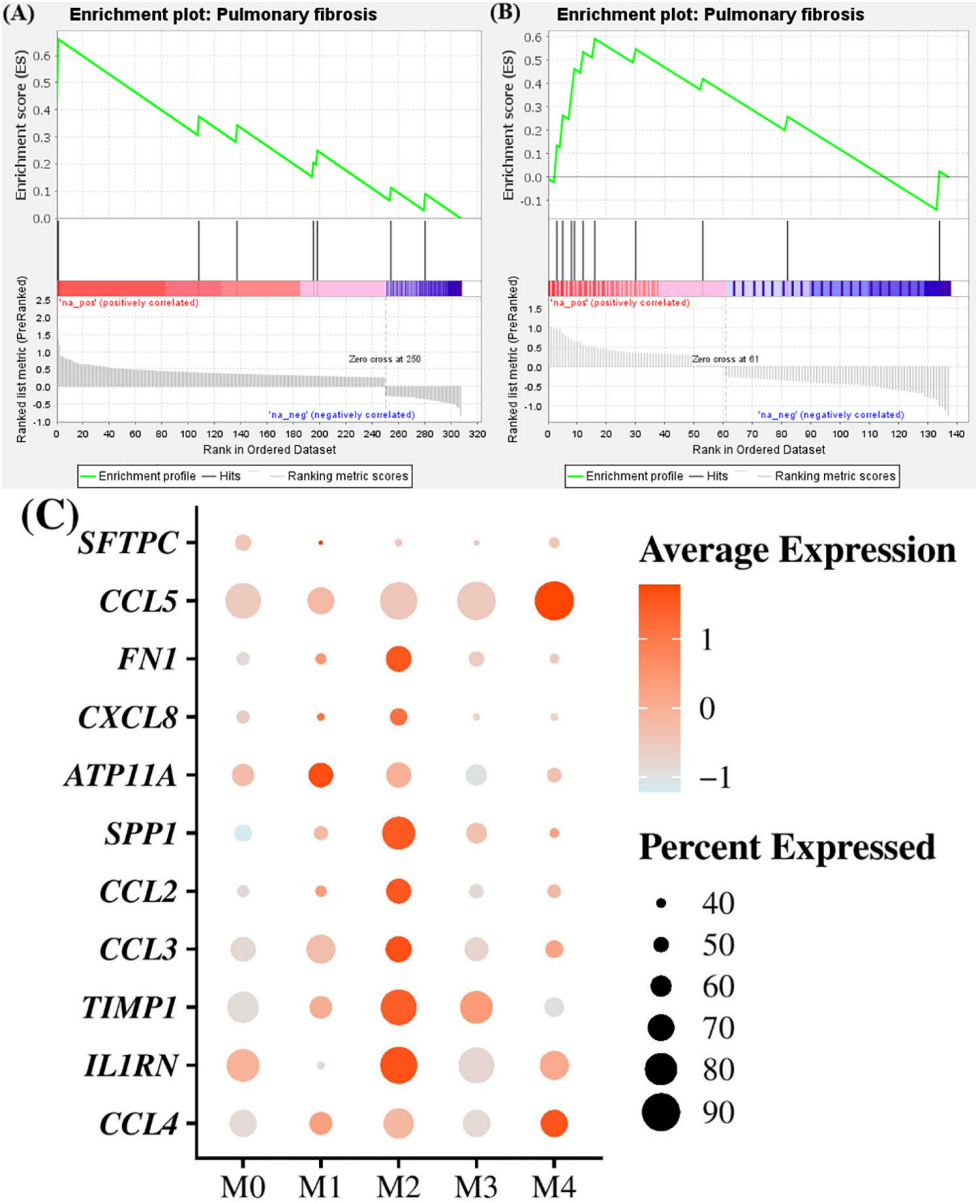
Enrichment in pulmonary fibrosis processes in M1 and M2 macrophages/monocytes clusters compared to others. **(A, B)** Gene set enrichment analyses between Comparative Toxicogenomics Database Pulmonary Fibrosis gene set and differentially expressed genes in M1 and M2 clusters, respectively, compared to others. **(C)** Dot plot showing the expression of genes involved in pulmonary fibrosis processes found to be upregulated in cluster M1 and M2 compared to others. Dot size represents the percentage of cells expressing the genes, while the dot color represents the average expression of the indicated genes.

For each cluster independently of the animal status (healthy or affected with CIPF), we also performed a GSEA to determine whether overexpressed genes in each cluster, in comparison with other clusters, could be associated with signatures of pulmonary fibrosis using the Comparative Toxicogenomics Database Pulmonary fibrosis gene set (24). Only cells in cluster M1 and M2 showed significant enrichment for pulmonary fibrosis with a normalized enrichment score (NES) of 1.87 and 1.85, respectively (FDR *q*-value = 0.007 and 0.002) (**Figures 4A, B**). Differentially overexpressed genes identified in relation with pulmonary fibrosis included *SFTPC* (encoding surfactant protein C), *CCL5* (encoding C-C motif chemokine ligand 5), *FN1* (encoding fibronectin 1), *CXCL8* (encoding C-X-C motif chemokine ligand 8), *ATP11A* (encoding ATPase phospholipid transporting 11A) and *SPP1* (encoding osteopontin) in cluster M1 and *CCL2* (encoding C-C motif chemokine ligand 2), *SPP1*, *FN1*, *CCL3* (encoding C-C motif chemokine ligand 3), *TIMP1* (encoding metallopeptidase inhibitor 1), *IL1RN* (encoding interleukin 1 receptor antagonist), *CXCL8* and *CCL4* (encoding C-C motif chemokine ligand 4) in cluster M2 (**Figure 4C**). M0 cells were negatively enriched for pulmonary fibrosis with a NES of −2.04 (FDR *q*-value = 0.002). The other clusters were not significantly associated with pulmonary fibrosis processes (FDR *q*-value = 0.145 and 0.289 for cells of cluster M3 and M4, respectively).

#### Comparison Between Animal Status

**Figure 5:**
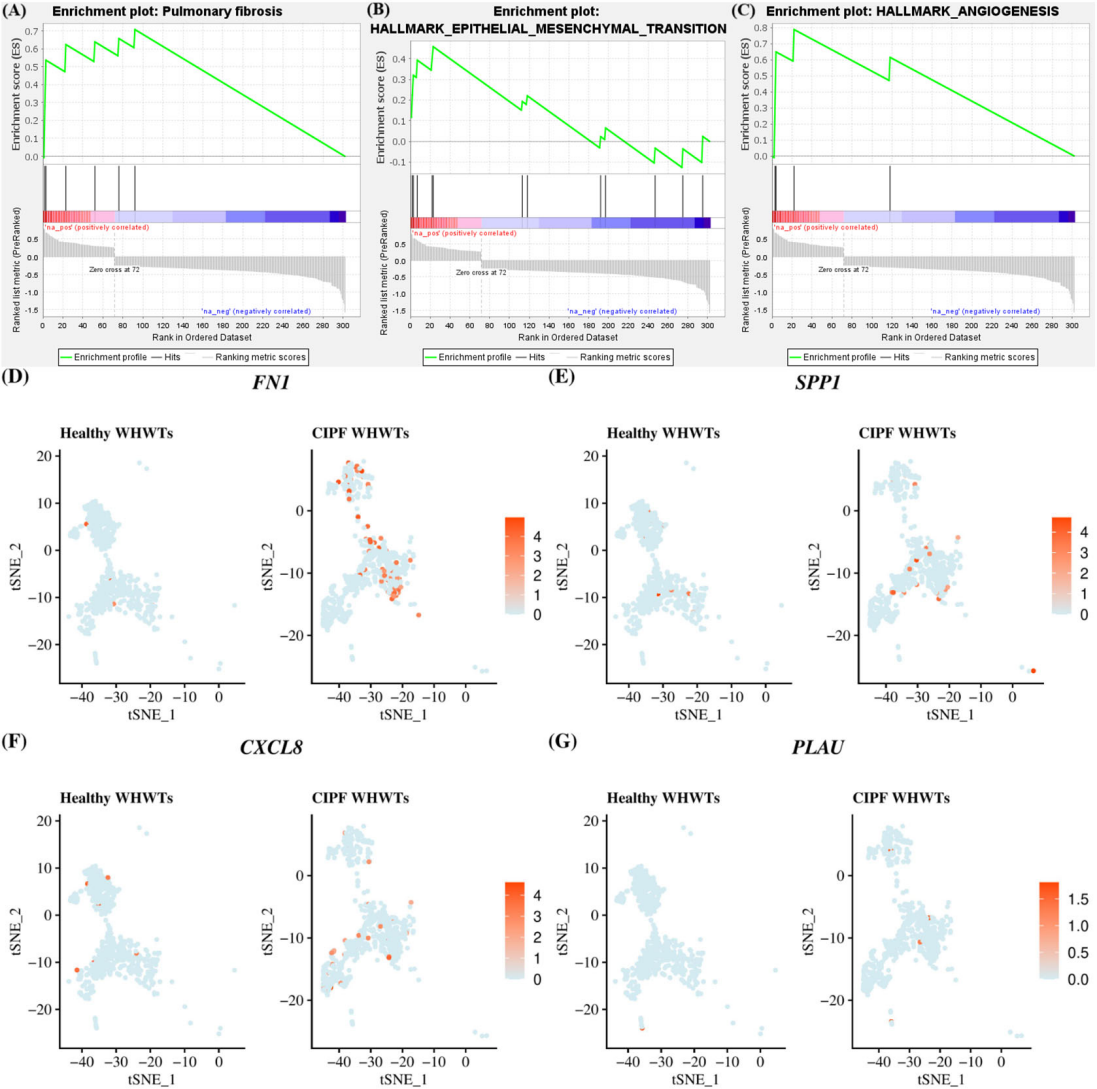
M1 macrophages/monocytes cluster enrichment in pulmonary fibrosis processes in CIPF compared with healthy dogs. **(A–C)** Gene set enrichment analyses in M1 cluster between differentially expressed genes in West Highland white terriers (WHWTs) affected with canine idiopathic pulmonary fibrosis (CIPF) compared to healthy WHWTs and the Comparative Toxicogenomics Database Pulmonary fibrosis gene set and epithelial mesenchymal transition and angiogenesis Hallmark gene sets. **(D–G)** T-distributed stochastic neighbor embedding (t-SNE) plot of cluster M1 cells showing overexpressed genes in CIPF compared with healthy WHWTs, associated with pulmonary fibrosis according to the Comparative Toxicogenomics Database Pulmonary fibrosis gene set. Color represents the average expression of the indicated genes.

Differential gene expression between cells from healthy WHWTs and WHWTs affected with CIPF in each Ma/Mo cluster was also assessed (**Supplementary Table 6**) and was essentially found for cells in cluster M1. DEGs in cluster M1 between CIPF and healthy WHWTs were mapped to the Comparative Toxicogenomics Database Pulmonary fibrosis gene set to assess pulmonary fibrosis signatures. NES in pulmonary fibrosis processes was at 2.01 (FDR *q*-value = 0.008) (**Figure 5A**). Genes involved in pulmonary fibrosis processes found to be upregulated in CIPF compared with healthy WHWTs in cluster M1 included *FN1*, *SPP1*, *CXCL8*, and *PLAU* (encoding plasminogen activator urokinase) (**Figures 5D–G**). The differential expression between healthy and CIPF WHWTs of those molecules in all Ma/Mo clusters is illustrated in **Supplementary Figure 2**. Moreover, in cluster M1, enrichment analysis with Hallmark gene sets indicated that cells from CIPF WHWTs were enriched for processes known to be associated with fibrosis including “epithelial mesenchymal transition (EMT)” (**Figure 5B**) and “angiogenesis” (**Figure 5C**) (NES of 1.86 and 1.88; FDR *q*-value = 0.039 and 0.068, respectively). Genes associated with these two gene sets and overexpressed in CIPF dogs included *VIM* (encoding vimentin), *FN1*, *SPP1*, *THY1* (encoding Thy-1 cell surface antigen, *CD90*) for “EMT” gene set and *SPP1*, *VCAN* (encoding large fibroblast proteoglycan) and *S100A4* (encoding S100 calcium binding protein A4) for “angiogenesis” gene set (**Supplementary Table 6**).

## Discussion

In this study, we analyzed Ma/Mo clusters in the BALF from healthy WHWTs compared with WHWTs affected with CIPF. Five Ma/Mo clusters were identified. Among them, we described a cluster of monocytes present in larger proportion in CIPF WHWTs than in healthy WHWTs. Expression of cells in this cluster was enriched for pulmonary fibrosis processes and eight genes associated with fibrosis were overexpressed in this cluster including *CCL2*, *SPP1*, *FN1*, *CCL3*, *TIMP1*, *IL1RN*, *CXCL8*, and *CCL4.* We also identified a cluster of monocyte-derived macrophages enriched for inflammatory and pulmonary fibrosis processes in which the gene expression differed between CIPF and healthy WHWTs with an enrichment for pulmonary fibrosis but also EMT and angiogenesis processes. We identified four overexpressed genes associated with pulmonary fibrosis processes in CIPF compared with healthy dogs in this cluster including *FN1*, *SPP1*, *CXCL8*, and *PLAU*.

In this study, similar cell populations and clusters were identified compared with previously published data on scRNA-seq analysis in BALFs from healthy dogs and included Ma/Mo, T cells either CD8^+^ or CD8^−^CD4^−^, DCs either mature or immature, neutrophils, B cells, epithelial cells, mast cells, and cycling cells (18). We were not able to differentiate between ciliated and non-ciliated epithelial cells which can be due to either the low proportion or the absence of ciliated epithelial cells in our samples (37) as rare cell populations may be missed using scRNA-seq (15). As already reported (18), eosinophils were not identified using scRNA-seq, probably secondary to their RNase content conducting to the degradation of mRNAs in those cells (38).

In healthy conditions, lung macrophages are known to be extremely heterogeneous and play a crucial role in the regulation of the homeostasis of the lung. In addition to their immune defense function, they also exerted an indispensable role in organ development, maintenance of homeostasis and repair (3, 17). In the lung, the majority of the macrophages are AMs which are resident and self-renewing macrophages (3). They have been identified in this study by their expression of *MARCO* and corresponded to cells of cluster M0 and M3 (3, 18, 26). In inflammatory conditions, the lung is rapidly infiltrated by recruited monocytes which gradually differentiate into monocyte-derived macrophages and then AMs (3). Here, we observed a higher proportion of monocytes (cells from cluster M2) in CIPF dogs that are probably recruited secondary to lung fibrosis in higher proportion than in healthy dogs. This increased number of macrophages and myeloid cells was also reported as an early event in bleomycin-induced lung fibrosis mouse model (6). As M2 cluster cells were enriched in pulmonary fibrosis processes, we suggest that their increased proportion in CIPF condition could participate to the onset and/or to the perpetuation of the fibrosis process in WHWTs.

The Ma/Mo involved in pro-fibrotic processes in this study can be considered as immature macrophages as they were identified as either monocyte-derived macrophages (cluster M1) or monocytes (cluster M2). Recently, transcriptomic proﬁling of macrophages collected over the time course of bleomycin induced ﬁbrosis showed that during monocyte maturation, genes linked to ﬁbrosis are most highly expressed during their differentiation and progressively downregulated with the maturation of the cells into AMs (39). This is in line with results obtained in this study and suggests that recently recruited macrophages (clusters M1 and M2) have greater ﬁbrotic capacity than mature AMs (clusters M0 and M3). Targeting those particularly pro-fibrotic recruited immature macrophage clusters could be a potential novel strategy for the prevention and the therapy of CIPF.

DEGs between healthy and CIPF WHWTs were essentially found in the M1 cluster. Moreover, M1 cells in CIPF dogs were enriched for EMT, angiogenesis and pulmonary fibrosis processes. EMT is considered as one of the phenomena by which collagen-producing fibroblasts and myofibroblasts accumulate, creating a pro-fibrotic environment (40). Indeed, epithelial cells differentiate to acquire features of mesenchymal cells including invasion, migration, and production of extracellular matrix (40). Altered EMT process is the most widely accepted pathogenetic mechanism in IPF patient (40) and could also participate in the development of CIPF as suggested by this study. Angiogenesis is another well-known mechanism involved in IPF, which is targeted by Nintedanib, an anti-angiogenesis molecule used in human for its properties against the vascular endothelial growth factor (VEGF) pathway (41). Involvement of angiogenesis in CIPF has only been assessed through the measurement of VEGF concentration in serum without results (42). To the authors’ best knowledge, none of the molecules identified in the present study and linked to angiogenesis has been studied in CIPF.

Among genes found to be associated with pulmonary fibrosis processes, only *CCL2* and *CXCL8* have already been associated with CIPF (2). Indeed, it has been shown that mRNA expression of *CXCL8* and *CCL2* was increased in CIPF lungs compared with controls (2). Moreover, *CCL2* and *CXCL8* chemokine concentrations were increased in CIPF WHWTs compared with healthy WHWTs in both serum and BALF and only in BALF respectively (2). The osteopontin (*SPP1* gene) is a glycoprotein secreted by numerous cell types including macrophages which has been proved to be closely related to IPF (7, 43–46). Indeed, high level of expression and increased BALF protein concentration have been reported in IPF mouse models but also in IPF patients (45). Such findings suggest that osteopontin could be used as a potential biomarker and a therapeutic target for treating fibrotic lung diseases (43). The fibronectin 1 (*FN1* gene) is a mediator of cell matrix adhesions. It promotes myofibroblast differentiation and is found in abundance in the lungs of IPF patients (47). *CCL3* and *CCL4*, also known as macrophage inflammatory protein 1-alpha and beta, are chemoattractant cytokines (48–50) suspected to play a role in sustaining inflammation and the chronic course of IPF by recruiting inflammatory cells such as neutrophils (49–51). Their expression in CIPF dogs could be related to the higher rate of neutrophils found in the BALF of CIPF compared to healthy dogs. The tissue inhibitor of metalloproteinase (*TIMP-1* gene) probably contributes, through its control of matrix metalloproteinase catalytic activity, to provide a non-degrading fibrillar collagen microenvironment in IPF patient as well as in IPF mouse model (52, 53). It has also a potential value as biomarker in patients with IPF (54). The interleukin-1 receptor antagonist (encoding *IL-1RA*) is a cytokine produced by alternatively activated AMs. The protein level was increased in IPF patients compared with healthy volunteers (55, 56) and in patients with acute exacerbation of IPF compared with stable IPF patients suggesting that this protein could be of interest as diagnostic and prognostic marker (56). The role of the plasminogen activator urokinase (*PLAU* gene) in pulmonary fibrosis is not clear. The protein level has been showed to be low in BALF of IPF patient (57, 58) and the molecule was showed to be protective against fibrosis development in IPF mouse model (59). Recently, the protective role of the plasminogen activator was controverted as its presence was associated with increased plasmin formation which in turn activates structural and inflammatory cells driven fibrosis (57). *PLAU* overexpression in this study indicates that fibrinolytic processes are present in CIPF dogs. Whether it is protective or not remains unclear. Further studies are needed to better assess the potential role of all these molecules in CIPF pathogenesis and their utility as biomarkers of disease progression and as potential therapeutic target.

The present study had some limitations. First, the analysis of scRNA-seq data remains limited by the poor annotation of canine genomic dataset highlighting the need for further studies to optimize the use of this technique in healthy and diseased dogs. Indeed, the percentage of reads mapped confidently to the transcriptome had to be from at least 30%^1^, which is not the case in this study. Secondly, our study involved a relatively low number of dogs either healthy or affected with CIPF. Indeed, even if the transcriptomic profiling costs are falling, the use of the scRNA-seq remains currently quite expensive. However, even with this small number of subjects and with the lack of annotation of the canine genome, we were able to identify the different cell populations, their genes expression and their DEGs in CIPF condition. We were also able to detect the genes already identified as involved in CIPF such as *CXCL8* and *CCL2*. Finally, it should be noted that in some Ma/Mo clusters, DEGs included markers normally expressed by other cell types, mainly in M4 cluster which expressed Ma/Mo and T cells markers. This likely results from contamination from ambient RNA released during BALF processing. This contamination is a known limitation that can occur in scRNA-seq experiments (60, 61). Another explanation would be that these cells are in fact doublets. Doublets are a known confounding factor in scRNA-seq analysis (62) that can be reduced by decreasing cell number introduced in the Chromium^TM^ Controller (63) and by filtering out cells with a really high gene count (62) as it was done in this study.

## Conclusion

Using scRNA-seq in BALF specimens from healthy WHWTs and WHWTs affected with CIPF, we were able to reveal the presence of pro-fibrotic monocytes, more abundant in CIPF than in healthy WHWTs, reflecting the inflammation that occurs in fibrotic lung. The presence of those monocytes enriched with pro-fibrotic genes probably participates to the onset and/or the perpetuation of CIPF in WHWTs. Moreover, monocyte-derived macrophages enriched in pro-fibrotic genes in CIPF compared with healthy WHWTs were also identified. This cluster was also enriched with EMT and angiogenesis processes, which are known to play an important role in IPF.

The results of that study offer promise for the better understanding of the role of macrophages in CIPF pathogenesis and the identification of new biomarkers and therapeutic targets to better diagnose, follow and treat the disease.

## Data Availability Statement

The datasets presented in this study can be found in online repositories. The names of the repository/repositories and accession number(s) can be found below: https://www.ebi.ac.uk/arrayexpress/, E-MTAB-9623.

## Ethics Statement

The animal study was reviewed and approved by the ethical committee of the University of Liège (approval no. 1435). All dogs were privately owned. Written informed consent was obtained from the owners for the participation of their animals in this study.

## Author Contributions

AF, CC, and FB designed the study. AF and CC recruited the dogs and collected samples. A-CM performed and interpreted echocardiographies. GB performed and interpreted thoracic scans. A-CT performed anesthesia of the dogs. AF, DP, and LF processed and acquired the data from samples. AF and DP analyzed the data. FB, CD, and TM provided their expertise in lung immune cells and helped for data interpretation. AF wrote the manuscript. All authors contributed to the article and approved the submitted version.

## Funding

This work was supported by a grant from the “Fonds Spéciaux de la Recherche” from the University of Liège.

## Conflict of Interest

The authors declare that the research was conducted in the absence of any commercial or financial relationships that could be construed as a potential conflict of interest.

## Footnotes

1 https://support.10xgenomics.com/single-cell-gene-expression/software/pipelines/latest/troubleshooting#alerts (accessed March 16, 2020).

## Abbreviations

CIPF, Canine idiopathic pulmonary fibrosis; WHWT, West Highland white terrier; HRCT, High-resolution computed tomography; IPF, Idiopathic pulmonary fibrosis; ScRNA-seq, Single-cell RNA sequencing; AMs, Alveolar macrophages; BALF, Bronchoalveolar lavage fluid; Ma/Mo, Macrophages/monocytes; DC, Dendritic cell; 6MWD, 6-min walked distance; TCC, Total cell count; DCC, Differential cell count; GSEA, Gene set enrichment analysis; FDR, False discovery rate; GO, Gene ontology; DEG, Differentially expressed gene; Avg_log FC, Average log2 fold change; t-SNE, t-distributed stochastic neighbor embedding; NES, Normalized enrichment score; EMT, Epithelial mesenchymal transition; VEGF, Vascular endothelial growth factor.

## References

[1] Laurila HP Rajamäki MM Update on canine idiopathic pulmonary fibrosis in West Highland white terriers. Vet Clin North Am - Small Anim Pract (2020) 50:431–46. 10.1016/j.cvsm.2019.11.004 31866093

[2] Clercx C Fastrès A Roels E Idiopathic pulmonary fibrosis in the West Highland white terrier: an update. Vet J (2018) 242:53–8. 10.1016/j.tvjl.2018.10.007 30503545

[3] Puttur F Gregory LG Lloyd CM Lloyd CM Fleming SA Airway macrophages as the guardians of tissue repair in the lung. Immunol Cell Biol (2019) 97:246–57. 10.1111/imcb.12235 30768869

[4] Aran D Looney AP Liu L Wu E Fong V Hsu A Reference-based analysis of lung single-cell sequencing reveals a transitional profibrotic macrophage. Nat Immunol (2019) 20:163–72. 10.1038/s41590-018-0276-y PMC6340744 30643263

[5] Reyfman PA Walter JM Joshi N Anekalla KR Mcquattie-pimentel AC Chiu S Single-cell transcriptomic analysis of human lung provides insights into the pathobiology of pulmonary fibrosis. Am J Respir Crit Care Med (2019) 199:1517–36. 10.1164/rccm.201712-2410OC PMC6580683 30554520

[6] Peyser R MacDonnell S Gao Y Cheng L Kim Y Kaplan T Defining the activated fibroblast population in lung fibrosis using single-cell sequencing. Am J Respir Cell Mol Biol (2019) 61:74–85. 10.1165/rcmb.2018-0313OC 30848683

[7] Morse C Tabib T Sembrat J Buschur KL Bittar HT Valenzi E Proliferating SPP1/MERTK-expressing macrophages in idiopathic pulmonary fibrosis. Eur Respir J (2019) 54:1802441. 10.1183/13993003.02441-2018 31221805 PMC8025672

[8] Xie T Wang Y Deng N Huang G Taghavifar F Geng Y Single-cell deconvolution of fibroblast heterogeneity in mouse pulmonary fibrosis. Cell Rep (2018) 22:3625–40. 10.1016/j.celrep.2018.03.010 PMC5908225 29590628

[9] Zhang Y Jiang M Nouraie M Roth MG Tabib T Winters S GDF15 is an epithelial-derived biomarker of idiopathic pulmonary fibrosis. Am J Physiol - Lung Cell Mol Physiol (2019) 317:510–21. 10.1152/ajplung.00062.2019 PMC6842909 31432710

[10] Gokey JJ Snowball J Sridharan A Speth JP Black KE Hariri LP MEG3 is increased in idiopathic pulmonary fibrosis and regulates epithelial cell differentiation. JCI Insight (2018) 3:e122490. 10.1172/jci.insight.122490 PMC6171798 30185671

[11] Xu Y Mizuno T Sridharan A Du Y Guo M Tang J Single-cell RNA sequencing identifies diverse roles of epithelial cells in idiopathic pulmonary fibrosis. JCI Insight (2016) 1:e90558. 10.1172/jci.insight.90558 27942595 PMC5135277

[12] Joshi N Watanabe S Verma R Jablonski RP Chen C Cheresh P A spatially restricted fibrotic niche in pulmonary fibrosis is sustained by M-CSF/M-CSFR signalling in monocyte-derived alveolar macrophages. Eur Respir J (2020) 55:1900646. 10.1183/13993003.00646-2019 31601718 PMC6962769

[13] Tsukui T Sun KH Wetter JB Wilson-Kanamori JR Hazelwood LA Henderson NC Collagen-producing lung cell atlas identifies multiple subsets with distinct localization and relevance to fibrosis. Nat Commun (2020) 11:1920. 10.1038/s41467-020-15647-5 32317643 PMC7174390

[14] Poczobutt JM Eickelberg O Defining the cell types that drive idiopathic pulmonary fibrosis using single-cell. Am J Respir Crit Care Med (2019) 199:1454–6. 10.1164/rccm.201901-0197ED PMC6580670 30715901

[15] See P Lum J Chen J Ginhoux F. A Single-cell sequencing guide for immunologists. Front Immunol (2018) 9:2425. 10.3389/fimmu.2018.02425 30405621 PMC6205970

[16] Stuart T Satija R Integrative single-cell analysis. Nat Rev Genet (2019) 20:257–72. 10.1038/s41576-019-0093-7 30696980

[17] Ji J Fan J Discovering myeloid cell heterogeneity in the lung by means of next generation sequencing. Mil Medecal Res (2019) 6:33. 10.1186/s40779-019-0222-9 PMC6814050 31651369

[18] Fastrès A Pirottin D Fievez L Marichal T Desmet CJ Bureau F Characterization of the bronchoalveolar lavage fluid by single cell gene expression analysis in healthy dogs: a promising technique. Front Immunol (2020) 11:1707. 10.3389/fimmu.2020.01707 32849601 PMC7406785

[19] Heikkila-Laurila HP Rajamaki MM Idiopathic pulmonary fibrosis in West Highland white terriers. Vet Clin Small Anim (2014) 44:129–42. 10.1016/j.cvsm.2013.08.003 24268338

[20] Schyns J Bai Q Ruscitti C Radermecker C De Schepper S Chakarov S Non-classical tissue monocytes and two functionally distinct populations of interstitial macrophages populate the mouse lung. Nat Commun (2019) 10:3964. 10.1038/s41467-019-11843-0 31481690 PMC6722135

[21] Cunningham F Achuthan P Akanni W Allen J Amode MR Armean IM Ensembl 2019. Nucleic Acids Res (2019) 47:745–51. 10.1093/nar/gky1113 PMC6323964 30407521

[22] Stuart T Butler A Hoffman P Hafemeister C Papalexi E Mauck III WM Comprehensive integration of single-cell data. Cell (2019) 177:1888–902. 10.1016/j.cell.2019.05.031 PMC6687398 31178118

[23] Subramanian A Tamayo P Mootha VK Mukherjee S Ebert BL Gillettea MA Gene set enrichment analysis: A knowledge-based approach for interpreting genome-wide expression profiles. Proc Natl Acad Sci (2005) 102:15545–50. 10.1073/pnas.0506580102 PMC1239896 16199517

[24] Davis AP Grondin CJ Johnson RJ Sciaky D McMorran R Wiegers J The comparative toxicogenomics database: update 2019. Nucleic Acids Res (2019) 47:948–54. 10.1093/nar/gky868 PMC6323936 30247620

[25] Gibbings SL Thomas SM Atif SM Mccubbrey AL Desch AN Danhorn T Three Unique interstitial macrophages in the murine lung at steady state. Am J Respir Cell Mol Biol (2017) 57:66–76. 10.1165/rcmb.2016-0361OC 28257233 PMC5516280

[26] Gibbings SL Goyal R Desch AN Leach SM Prabagar M Atif SM Transcriptome analysis highlights the conserved difference between embryonic and postnatal-derived alveolar macrophages. Blood (2015) 126:1357–66. 10.1182/blood-2015-01-624809 PMC4566811 26232173

[27] Gautier EL Shay T Miller J Greter M Jakubzick C Ivanov S Gene-expression profiles and transcriptional regulatory pathways that underlie the identity and diversity of mouse tissue macrophages. Nat Immunol (2012) 13:1118–28. 10.1038/ni.2419 PMC3558276 23023392

[28] Yu YRA Hotten DF Malakhau Y Volker E Ghio AJ Noble PW Flow cytometric analysis of myeloid cells in human blood, bronchoalveolar lavage, and lung tissues. Am J Respir Cell Mol Biol (2016) 54:13–24. 10.1165/rcmb.2015-0146OC 26267148 PMC4742930

[29] Bharat A Bhorade S Morales-Nebreda L McQuattie-Pimentel A Soberanes S Ridge K Flow cytometry reveals similarities between lung macrophages in humans and mice. Am J Respir Cell Mol Biol (2016) 54:147–9. 10.1017/CBO9781107415324.004 PMC4742931 26274047

[30] Byrne AJ Powell JE Sullivan BJO Ogger PP Hoffland A Cook J Dynamics of human monocytes and airway macrophages during healthy aging and after transplant. J Exp Med (2020) 217:e20191236. 10.1084/jem.20191236 31917836 PMC7062517

[31] Stifano G Christmann RB Macrophage involvement in systemic sclerosis: do we need more evidence? Curr Rheumatol Rep (2016) 18:2. 10.1007/s11926-015-0554-8 26700912

[32] Gundra UM Girgis NM Ruckerl D Jenkins S Ward LN Kurtz ZD Alternatively activated macrophages derived from monocytes and tissue macrophages are phenotypically and functionally distinct. Blood (2014) 123:110–22. 10.1182/blood-2013-08-520619 PMC4023427 24695852

[33] Ammon C Meyer SP Schwarzfischer L Krause SW Andreesen R Kreutz M Comparative analysis of integrin expression on monocyte-derived macrophages and monocyte-derived dendritic cells. Immunology (2000) 100:364–9. 10.1046/j.1365-2567.2000.00056.x PMC2327027 10929059

[34] Croxford AL Lanzinger M Hartmann FJ Schreiner B Mair F Pelczar P The cytokine GM-CSF drives the inflammatory signature of CCR2+ monocytes and licenses autoimmunity. Immunity (2015) 43:502–14. 10.1016/j.immuni.2015.08.010 26341401

[35] Patel VI Metcalf JP Airway macrophage and dendritic cell subsets in the resting human lung. Crit Rev Immunol (2019) 38:303–31. 10.1615/CritRevImmunol.2018026459 PMC6392073 30806245

[36] Alcover A Alarcon B Bartolo V Di. Cell biology of T-cell receptor expression and regulation. Annu Rev Immunol (2018) 36:103–25. 10.1146/annurev-immunol042617-053429 29261409

[37] Nelson WR Couto CG Diagnostic tests for the lower respiratory tract. In: Nelson WR Couto CG, editors. Small animal internal medicine, 6th ed St Louis, MI: Elsevier (2020). p. 287–320.

[38] Sattasathuchana P Steiner M Canine eosinophilic gastrointestinal disorders. Anim Heal Res Rev (2014) 15:76–86. 10.1017/S1466252314000012 24815742

[39] Misharin AV Morales-Nebreda L Reyfman PA Cuda CM Walter JM McQuattie-Pimentel AC Monocyte-derived alveolar macrophages drive lung fibrosis and persist in the lung over the life span. J Exp Med (2017) 214:2387–404. 10.1084/jem.20162152 PMC5551573 28694385

[40] Salton F Volpe MC Confalonieri M Epithelial-mesenchymal transition in the pathogenesis of idiopathic pulmonary fibrosis. Medicina (2019) 55:83. 10.3390/medicina55040083 PMC6524028 30925805

[41] Rivera-ortega P Hayton C Blaikley J Leonard C Chaudhuri N Nintedanib in the management of idiopathic pulmonary fibrosis: clinical trial evidence and real-world experience. Ther Adv Respir Dis Rev (2018) 12:1753466618800618. 10.1177/1753466618800618 PMC6156214 30249169

[42] Roels E Krafft E Antoine N Farnir F Laurila HP Holopainen S Evaluation of chemokines CXCL8 and CCL2, serotonin, and vascular endothelial growth factor serum concentrations in healthy dogs from seven breeds with variable predisposition for canine idiopathic pulmonary fibrosis. Res Vet Sci (2015) 101:57–62. 10.1016/J.RVSC.2015.05.020 26267090

[43] Dong J Ma Q Osteopontin enhances multi-walled carbon nanotube-triggered lung fibrosis by promoting TGF- β1 activation and myofibroblast differentiation. Part Fibre Toxicol (2017) 14:18. 10.1186/s12989-017-0198-0 28595626 PMC5465601

[44] Berman JS Serlin D Li X Whitley G Hayes J Rishikof DC Altered bleomycin-induced lung fibrosis in osteopontin-deficient mice. Am J Physiol - Lung Cell Mol Physiol (2004) 286:1311–8. 10.1152/ajplung.00394.2003 14977630

[45] Pardo A Gibson K Cisneros J Richards TJ Yang Y Becerril C Up-regulation and profibrotic role of osteopontin in human idiopathic pulmonary fibrosis. PloS Med (2005) 2:e251. 10.1371/journal.pmed.0020251 16128620 PMC1198037

[46] Wang H Wang M Xiao K Zhang X Wang P Qi H Bioinformatics analysis on differentially expressed genes of alveolar macrophage in IPF. Exp Lung Res (2019) 45:288–96. 10.1080/01902148.2019.1680765 31762326

[47] Upagupta C Shimbori C Alsilmi R Kolb M Matrix abnormalities in pulmonary fibrosis. Eur Respir Rev (2018) 27:180033. 10.1183/16000617.0033-2018 29950306 PMC9489108

[48] Bhavsar I Miller CS Al-Sabbagh M Macrophage inflammatory protein-1 alpha (MIP-1 alpha)/CCL3: as a biomarker. In: Preedy VR Patel VB, editors. general methods in biomarker research and their applications. Dordrecht, The Netherlands (2015). p. 223–49. 10.1007/978-94-007-7696-8_27

[49] Capelli A Di Stefano A Gnemmi I Donner CF CCR5 expression and CC chemokine levels in idiopathic pulmonary fibrosis. Eur Respir J (2005) 25:701–7. 10.1183/09031936.05.00082604 15802346

[50] Lee J Arisi I Puxeddu E Mramba LK Amicosante M Swaisgood CM Bronchoalveolar lavage (BAL) cells in idiopathic pulmonary fibrosis express a complex pro-inflammatory, pro-repair, angiogenic activation pattern, likely associated with macrophage iron accumulation. PloS One (2018) 13:e0194803. 10.1371/journal.pone.0194803 29649237 PMC5896901

[51] Capelli A Stefano ADI Lusuardi M Gnemmi I Donner CF Increased macrophage inflammatory protein-1a and macrophage inflammatory protein-1b levels in bronchoalveolar lavage fluid of patients affected by different stages of pulmonary sarcoidosis. Am J Respir Crit Care Med (2002) 165:236–41. 10.1164/rccm2106084 11790661

[52] Pardo A Cabrera S Maldonado M Selman M Role of matrix metalloproteinases in the pathogenesis of idiopathic pulmonary fibrosis. Respir Res (2016) 17:23. 10.1186/s12931-016-0343-6 26944412 PMC4779202

[53] Selman M Ruiz V Cabrera S Segura L Ramirez R Barrios R TIMP-1, -2, -3, and -4 in idiopathic pulmonary fibrosis. A prevailing nondegradative lung microenvironment? Am J Physiol - Lung Cell Mol Physiol (2000) 279:562–74. 10.1152/ajplung.2000.279.3.L562 10956632

[54] Todd JL Vinisko R Liu Y Neely ML Overton R Flaherty KR Circulating matrix metalloproteinases and tissue metalloproteinase inhibitors in patients with idiopathic pulmonary fibrosis in the multicenter IPF-PRO Registry cohort. BMC Pulm Med (2020) 20:64. 10.1186/s12890-020-1103-4 32171287 PMC7071646

[55] Stahl M Schupp J Jag̈er B Schmid M Zissel G Mul̈ler-Quernheim J Lung collagens perpetuate pulmonary fibrosis via CD204 and M2 macrophage activation. PloS One (2013) 8:e81382. 10.1371/journal.pone.0081382 24278429 PMC3835428

[56] Schupp JC Binder H Jäger B Cillis G Zissel G Müller-quernheim J Macrophage activation in acute exacerbation of idiopathic pulmonary fibrosis. PloS One (2015) 10:e0116775. 10.1371/journal.pone.0116775 25590613 PMC4295887

[57] Schuliga M Grainge C Westall G Knight D The fibrogenic actions of the coagulant and plasminogen activation systems in pulmonary fibrosis. Int J Biochem Cell Biol (2018) 97:108–17. 10.1016/j.biocel.2018.02.016 29474926

[58] Günther A Mosavi P Ruppert C Heinemann S Temmesfeld B Velcovsky HG Enhanced tissue factor pathway activity and fibrin turnover in the alveolar compartment of patients with interstitial lung disease. Thromb Haemost (2000) 83:853–60. 10.1055/s-0037-1613933 10896238

[59] Navaratnam V Fogarty AW McKeever T Thompson N Jenkins G Johnson SR Presence of a prothrombotic state in people with idiopathic pulmonary fibrosis: A population-based case-control study. Thorax (2014) 69:207–15. 10.1136/thoraxjnl-2013-203740 24002055

[60] Zheng GXY Terry JM Belgrader P Ryvkin P Bent ZW Wilson R Massively parallel digital transcriptional profiling of single cells. Nat Commun (2017) 8:14049. 10.1038/ncomms14049 28091601 PMC5241818

[61] Hwang B Lee JH Bang D Single-cell RNA sequencing technologies and bioinformatics pipelines. Exp Mol Med (2018) 50:96. 10.1038/s12276-018-0071-8 30089861 PMC6082860

[62] Ilicic T Kim JK Kolodziejczyk AA Bagger FO McCarthy DJ Marioni JC Classification of low-quality cells from single-cell RNA-seq data. Genome Biol (2016) 17:29. 10.1186/s13059-016-0888-1 26887813 PMC4758103

[63] Bloom JD Estimating the frequency of multiplets in single-cell RNA sequencing from cell-mixing experiments. PeerJ (2018) 6:e5578. 10.7717/peerj.5578 30202659 PMC6126471

