# Supplementary material for: Identification of Pro-Fibrotic Macrophage Populations by Single-Cell Transcriptomic Analysis in West Highland White Terriers Affected With Canine Idiopathic Pulmonary Fibrosis
Source: Front Immunol. 2020 Dec 15;11:611749. doi: 10.3389/fimmu.2020.611749 (PMC7770158; doi:10.3389/fimmu.2020.611749)
Supplement: Supplementary file 7 [file DataSheet_1.docx]

Supplementary Figures


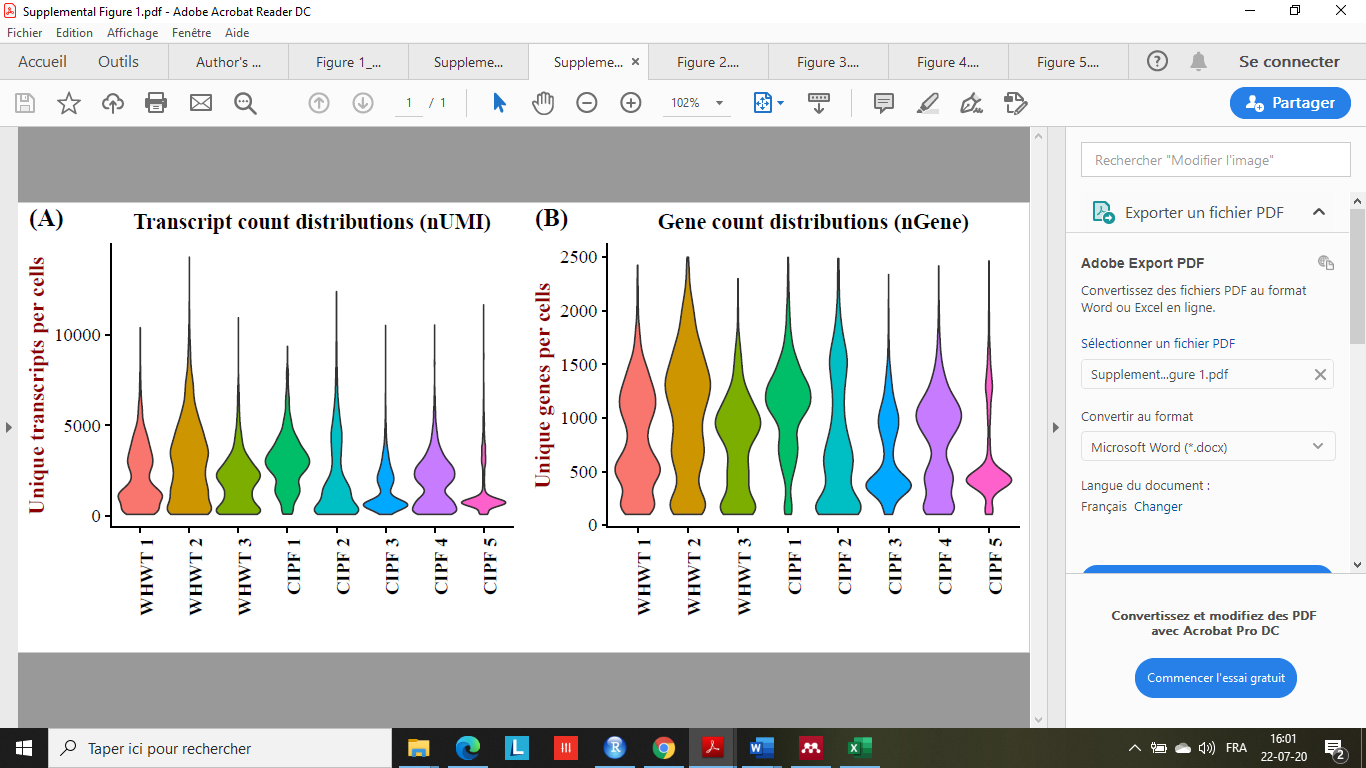


**Supplementary Figure 1.** **Dataset quality control. (A)** Unique transcript (nUMI) distributions; **(B)** Unique gene (nGene) distributions. Colors match across dogs’ bronchoalveolar lavage fluid samples. WHWT 1-3, samples from healthy West Highland white terriers; CIPF 1-5, samples from West Highland white terriers affected with canine idiopathic pulmonary fibrosis.


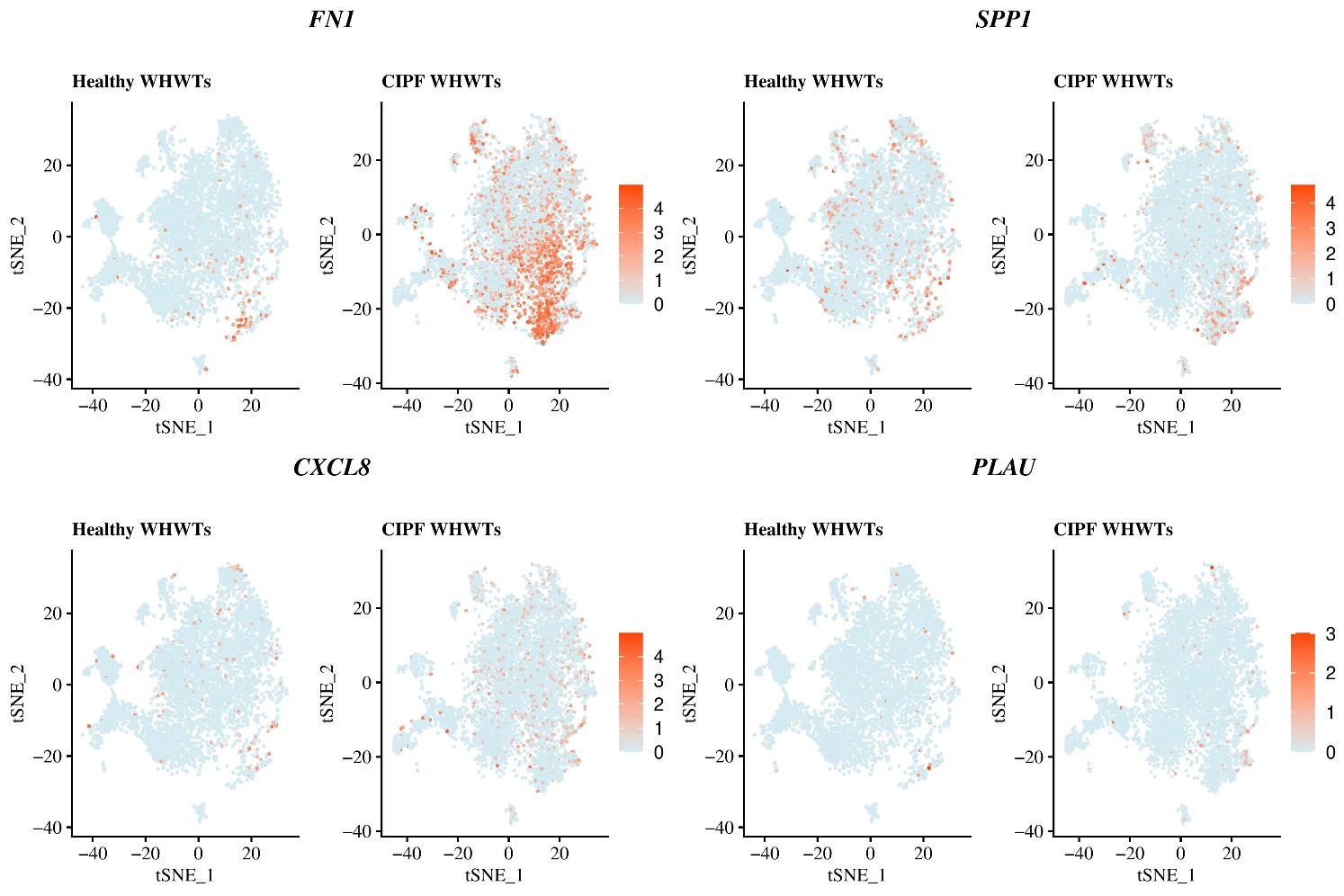


**Supplementary Figure 2. Pro-fibrotic upregulated genes between CIPF and healthy WHWTs in cluster M1.** T-distributed stochastic neighbor embedding (t-SNE) plot of all macrophages/monocytes clusters showing overexpressed genes associated with pulmonary fibrosis according to the Comparative Toxicogenomics Database Pulmonary fibrosis gene set between CIPF and healthy WHWTs in cluster M1. Color represents the average expression of the indicated genes.
